# Advances and Drawbacks of Sous-Vide Technique—A Critical Review

**DOI:** 10.3390/foods13142217

**Published:** 2024-07-15

**Authors:** Georgiana Ancuta Misu, Cristina Maria Canja, Mirabela Lupu, Florentina Matei

**Affiliations:** 1 Faculty of Biotechnology, University of Agronomic Sciences and Veterinary Medicine of Bucharest, 59 Marasti Boulevard, District 1, 011464 Bucharest, Romania; misu.anca@yahoo.com (G.A.M.); florentina.matei@unitbv.ro (F.M.); 2 Faculty of Food Industry and Tourism, Transilvania University of Brașov, 148 Castelului St., 500014 Brasov, Romania; lupu.mirabela@unitbv.ro

**Keywords:** sous-vide, food safety, nutritional retention, quality parameters

## Abstract

The sous-vide (SV) technique, notable for its precision and ability to preserve food quality, has become a transformative method in culinary arts. This review examines the technical aspects, applications, and limitations of SV, focusing on its impact on food safety, nutritional retention, and quality parameters across various food matrices such as meats, seafood, vegetables, and semi-prepared products. Through an extensive literature review, the study highlights the use of natural inhibitors and essential oils to enhance microbial safety and explores the nutritional benefits of SV in preserving vitamins and minerals. The findings suggest that while SV offers significant benefits in terms of consistent results and extended shelf life, challenges remain in terms of equipment costs and the necessity for specific training, and although sufficient for food preparation/processing, its effectiveness in eliminating microbial pathogens, including viruses, parasites, and vegetative and spore forms of bacteria, is limited. Overall, the research underscores SV’s adaptability and potential for culinary innovation, aligning with modern demands for food safety, quality, and nutritional integrity.

## 1. Introduction

Sous-vide, a French term for “under vacuum”, is a culinary method that has transformed modern cooking by marrying precise scientific techniques with traditional culinary art. This method involves vacuum-sealing food and cooking it in a water bath at meticulously controlled temperatures, renowned for preserving the food’s integrity and enhancing its flavor [1]. Sous-vide cooking has evolved from a specialty technique in gourmet kitchens to a globally recognized culinary practice.

The initial development of SV is attributed to American and French chefs’ efforts to improve cooking consistency and quality. This technique gained prominence through advancements in food science, making it versatile and globally recognized. In the past recent years, new research paths have been dedicated to this technique. A significant advancement in SV cooking is the integration of natural inhibitors and essential oils. The use of natural inhibitors, such as oregano and citric acid, to increase the thermal sensitivity of bacteria in salmon, as researched by Dogruyol et al. [2], and the investigation into the nutritional values of potato slices with rosemary essential oil by Amoroso et al. [3], show the method’s adaptability and potential for culinary experimentation. Further research demonstrated the antimicrobial effects of thyme and rosemary essential oils against *Listeria monocytogenes* in SV turkey and rainbow trout [4,5,6]. Öztürk et al. [7] investigated the impact of laurel and basil essential oils on the oxidative stability of sea bass fillets, while other researchers [8,9] explored the effectiveness of sage essential oil against pathogens in beef. Furthermore, two studies, conducted in 2023 by Lu et al. [10] and Hobani et al. [11], reinforce SV’s effectiveness in ensuring food safety and enhancing the sensory qualities of meats, while other authors [12] explored the combined effect of high hydrostatic pressure, SV cooking, and carvacrol on the quality of veal, illustrating the technique’s compatibility with other food processing methods.

The nutritional aspect of SV cooking is significant, as the method preserves vitamins and minerals often lost in traditional cooking methods. This holds significant importance for health-conscious consumers and environments where maintaining nutritional value is of utmost importance. These findings are supported by research conducted in 2024 by Mayurnikova et al. [13] on the impact of traditional and modern technologies, including SV cooking, on preserving the nutritional value of semi-finished food products. Additionally, the vacuum-sealing process in SV cooking inhibits bacterial growth, thus extending the shelf life of food products. This is particularly relevant for reducing food waste and enhancing food safety in global food distribution and storage. Kaya et al. [14] explore this concept further by examining the effects of different packaging methods, including sous-vide, on the chemical, sensory, and microbiological qualities of SV-cooked foods.

The same research conducted by Kaya et al. [14] shows SV cooking continues to evolve; it stands as a symbol of culinary innovation, blending tradition with modern technology. The method’s impact on food safety, nutritional preservation, and culinary creativity makes it a significant development in modern gastronomy and food science. Sous-vide cooking, with its emphasis on precision, flavor enhancement, and nutritional preservation, is redefining the culinary landscape, offering new possibilities for creating high-quality, nutritious, and flavourful dishes [6].

Recent research offers insights into the evolving perceptions and applications of SV technology. For instance, the study from 2022 by Avató et al. [1] on consumer preferences for ready-to-eat SV food products elucidates a growing consumer inclination towards high-quality, convenient meal options that do not sacrifice sensory or nutritional value. This signals a shift towards premium, ready-to-consume food solutions that resonate with the fast-paced lifestyle of contemporary consumers. Similarly, another investigation into the development of plant-based, ready-to-eat dishes utilizing SV technology reveals an innovative approach to augment vegetable consumption [15]. This aligns with current dietary trends, offering gastronomically appealing and nutritionally enhanced vegetable preparations, thereby highlighting SV’s versatility in addressing modern dietary preferences.

The novelty of this review is underscored by its comprehensive scope and critical approach, distinguishing it from recent reviews on SV cooking. Previous works, such as that of Kathuria et al. [16], primarily focus on the technological and functional properties of SV but fail to delve deeply into its adaptability across various food matrices. This review bridges that gap by exploring SV applications from small kitchens to industrial settings, emphasizing its potential for culinary experimentation.

Similarly, the review by Singh et al. [17] concentrates on SV’s impact on meat products, often neglecting its effects on vegetables and semi-prepared foods. Our review addresses this limitation by examining the nutritional and sensory impacts of SV on a wider range of foods, including vegetables and seafood.

The study from 2021 by Cui et al. [18] highlights the benefits and drawbacks of SV but does not critically assess the integration of natural inhibitors for food safety. This review goes further by analyzing how natural inhibitors and essential oils can enhance microbial safety in SV cooking, thus extending its practical applications.

Lastly, research conducted by Latoch et al. [19] focuses predominantly on meat without sufficiently discussing SV’s nutritional benefits and its role in extending shelf life. This review fills that void by examining how SV preserves vitamins and minerals, thereby contributing to reduced food waste and better food safety.

This review offers an expansive and critical evaluation of SV cooking, addressing its applications, benefits, and limitations across various food types and incorporating modern advancements for enhanced food safety and nutritional retention. It evaluates the nutritional retention and sensory qualities across various food matrices and explores technological advancements and challenges associated with sous-vide equipment in various environments. By integrating natural inhibitors and essential oils to enhance microbial safety, this review provides invaluable guidance for future research, culinary innovation, and industry practices, ensuring that the benefits of SV cooking are maximized while mitigating associated risks.

## 2. Methods

In our review, we employed a comprehensive methodology to explore the multifaceted aspects of SV cooking. The review strictly includes studies focusing on the application of sous-vide from domestic kitchens to industrial uses, emphasizing the technique’s impact on food safety, nutritional retention, and quality parameters such as texture and flavor. To capture a broad spectrum of relevant literature, we conducted an exhaustive search across several databases, including PubMed, Web of Science, and Scopus, as well as specialized food science journals and gray literature up to the most recent year completed, ensuring a robust dataset.

Our search strategy was meticulously designed to include terms related to sous-vide cooking, its nutritional impacts, microbial safety, and sensory qualities. We applied specific filters to select studies from the last two decades, enhancing the review’s relevance and comprehensiveness. The selection process involved an initial screening of titles and abstracts by two independent reviewers, using predefined inclusion and exclusion criteria. Discrepancies were resolved through discussion, with a third reviewer available for unresolved conflicts.

Data extraction was carried out independently by two reviewers, focusing on variables directly relevant to the review’s questions, such as the type of sous-vide equipment used, cooking conditions, and outcomes related to safety, nutrition, and sensory attributes. We assessed the risk of bias in individual studies using a customized tool designed to address the unique challenges associated with evaluating culinary technology research.

For effect measures, we adapted metrics specific to each desired outcome, ranging from pathogen reduction levels for assessing food safety to nutrient retention rates for evaluating nutritional impacts. We planned both qualitative and quantitative syntheses, including meta-analyses where appropriate, to aggregate the findings and assess the overall effectiveness and impacts of sous-vide cooking.

## 3. Sous-Vide Cooking: Equipment and Innovative Solutions

Sous-vide refers to a culinary technique where food is vacuum-sealed in a plastic pouch and then cooked in a water bath at a precisely controlled temperature. This low-temperature cooking method is known for its unique ability to cook food evenly, ensuring that the inside is properly cooked without overcooking the outside and retaining moisture.

The SV method is distinguished by its meticulous temperature control [20]. The technique enhances the texture and flavor of food. The precise control of the cooking temperature allows for the perfect doneness of meat, tenderizing even the toughest cuts by breaking down fibers without losing moisture, however, bringing dismal improvements in eliminating microbial pathogens [21,22].

In terms of innovation, SV has opened doors to new culinary possibilities. Its combination with other cooking techniques, like marinating and grilling, has been explored to further enhance the sensory qualities of foods such as beef. These combinations can optimize tenderness and juiciness, offering a more palatable experience [23].

Sous-vide cooking is also recognized for its role in food safety and preservation. By cooking food at precise temperatures, SV can effectively eliminate harmful pathogens, making it a safe method for preparing various types of food. This aspect of SV is crucial for mass catering establishments where food safety is paramount [22].

Recent studies have made progress and highlighted SV as a viable approach for microbial food safety assurance. Unlike conventional thermal processing, which can significantly alter food quality and nutrition, SV’s precise nature preserves these aspects, even though its effectiveness in eliminating microbial pathogens, including viruses, parasites, and vegetative and spore forms of bacteria, is limited [24].

In addition to culinary applications, SV techniques have inspired innovations in other fields. For instance, a SV-inspired method has been developed for impregnating amorphous titanium (hydr)oxide into carbon block filters for arsenic removal from water, demonstrating the technique’s potential beyond the kitchen [25].

Furthermore, the comparison of SV with traditional hydrothermal treatments has shed light on its impact on glucosinolate (GLS) content in *Brassica* vegetables, highlighting the method’s ability to retain valuable nutrients [26]. Similarly, the effects of SV on the physical, microstructural, and antioxidative properties of pumpkin cubes have been studied, showcasing its benefits over conventional and vacuum cooking methods [27].

The use of SV technology in both home and catering settings has been examined for its impact on nutritional value and energy consumption. While SV cooking preserves the nutritional value of foods like chicken and reduces food waste, it is more energy-intensive compared to traditional cooking methods. This factor makes SV more suitable for foodservice applications than for home use [28].

Sous-vide cooking, characterized by its stringent temperature monitoring, relies on specialized equipment for both home cooks and professional chefs. The equipment’s range and sophistication vary depending on the setting, from basic home kitchens to industrial-scale food production.

**Figure 1:**
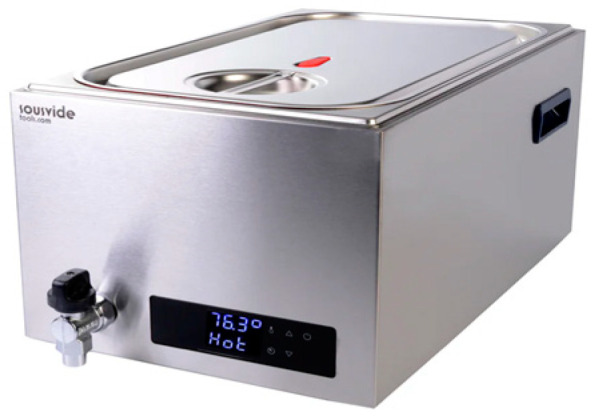
Laboratory SV equipment. (source: https://www.sousvidetools.com/sous-vide (accessed on 25 June 2024)).

**Domestic Cooking Equipment**: In the study conducted by Kodipelli et al. [29], the preferences of amateur chefs are highlighted, revealing a tendency towards the use of more accessible SV equipment, such as Ziplock bags or other non-vacuum-sealing methods, as opposed to advanced professional vacuum-sealing tools. This approach, though more user-friendly, is identified as possibly contributing to an increase in aerobic bacterial growth due to the inevitable presence of air. Additionally, Kodipelli et al. [29] delve into the exploration of alternative preparation methods, including the use of gas-fired surfaces and the application of dry salting to meat prior to vacuum sealing. This investigation aims to evaluate their impact on the microbiological safety and quality of beef prepared via SV, offering a comprehensive look at potential enhancements in SV cooking practices. This is similar to the equipment you find in small laboratories, as illustrated in Figure 1.

**Gastronomic Kitchen Equipment**: In gastronomic kitchens, the practice of SV cooking is elevated through the use of sophisticated equipment. Restaurants typically employ precise temperature-controlled water baths alongside high-quality vacuum sealers, setting a standard in the industry. Such advanced tools not only ensure uniform cooking outcomes but also significantly enhance the sensory attributes of a variety of meats. These technological advantages have been instrumental in solidifying SV’s esteemed position within the culinary realm [30]. Complementing this, a 2020 comprehensive review by Stankov et al. [31] underscored the pivotal role of SV in the restaurant industry. Their analysis delves into the sensory quality improvements attributed to SV cooking, highlighting how vacuum packaging and low-temperature cooking work synergistically to preserve nutritional value, improve texture and tenderness, and extend shelf life, further advocating for the method’s adoption across professional settings.

**Figure 2:**
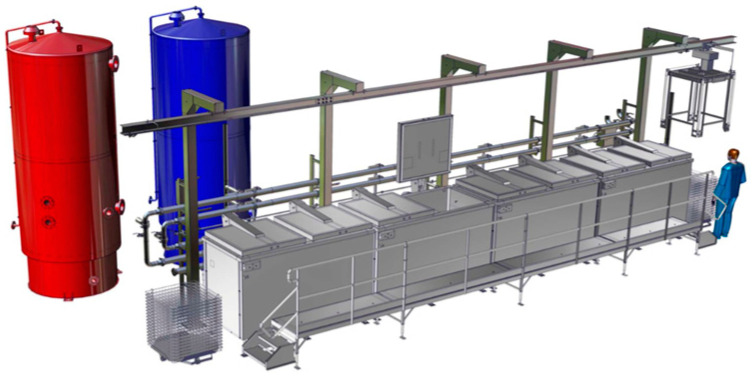
Armor Inox industrial line representation. Water tanks (red—hot water; blue—cold water) connected to cooking unit line (gray). (source: https://www.armorinox.com/en/equipment/thermix-sous-vide (accessed on 25 June 2024)).

**Industrial Sous-Vide Machinery**: On an industrial scale, producers of the likes of Armor Inox have created compact lines that allow for the production of SV products using cold and hot water tanks, which allow for the precise and rapid control of the water temperature in cooking vessels (vessels in front of the tanks), as can be seen in Figure 2. This incorporation of water baths has been engineered to ensure the uniform cooking of extensive quantities of food. Especially within the meat processing sector, industrial SV equipment plays a critical role, facilitating the production of a diverse array of products. This technology not only guarantees consistent cooking outcomes but also significantly prolongs the product shelf life, enhancing overall food safety and quality [32]. Expanding on this, Nosnova et al. [33] explore the application of SV technology in beef product manufacturing, highlighting its benefits in achieving optimal cooking precision and product shelf life. Similarly, Thathsarani et al. [34] provide further insights into the historical evolution and industrial utilization of SV in the meat industry. Their collective research underlines the vital contribution of SV technology in streamlining production lines, ensuring product uniformity, and addressing the demands of the contemporary food industry.

Nutrient retention is a hallmark of SV cooking, applicable to both meats and vegetables. This cooking method adeptly preserves vitamins and minerals that are often lost in conventional cooking methods. The vacuum-seal process minimizes nutrient depletion, and the gentle cooking temperatures safeguard heat-sensitive nutrients, aligning perfectly with health-conscious dietary trends. Moreover, SV cooking excels in enhancing food safety. The precise control over temperature facilitates effective pasteurization, especially in meats and fish, significantly reducing the risk of foodborne illnesses. This method is not just about preserving flavor and texture; it is about ensuring safety, as reinforced by studies like those of Redfern et al. [35] and Gál et al. [9]. Sous-vide cooking, therefore, presents itself as a comprehensive culinary solution, enhancing the safety, flavor, and texture of food.

## 4. Sous-Vide for Meat Processing

When it comes to meats, the SV method stands unmatched. Its ability to tenderize is profound—tough cuts like beef short ribs, horsemeat, and various pork cuts are rendered succulent and delectably tender. Unlike traditional cooking methods, where achieving the perfect balance of tenderness and moisture can be elusive, SV operates at the sweet spot of low temperatures and extended cooking times. This method meticulously breaks down the tough fibers without leaching out the natural juices, ensuring meats like chicken breast and beef tenderloin are not only deliciously tender but also perfectly safe, as indicated in studies by Karki et al. [36] and Noh et al. [37].

The structural integrity of meat, characterized by its muscular fibers and connective tissues, plays a pivotal role in determining its qualitative attributes. Uncooked meat showcases a pristine architectural framework, highlighted by cellular separations filled with air, facilitating the dissociation between cells, a phenomenon initially identified by Hong et al. [38]. Furthermore, the same research [38] showed that in contemporary culinary practices, consumer preferences have evolved, with monetary value being directly correlated with perceived meat quality, gauged through parameters such as coloration, succulence, tenderness, and aromatic qualities. Moreover, the importance of nutritional content and the assurance of product safety have emerged as fundamental considerations.

A prevalent belief amongst consumers posits that meat exhibiting a vibrant red hue is indicative of superior freshness as opposed to its brown-toned counterparts. Yet, fresh meat is susceptible to a trio of major detriments: microbial proliferation, lipid peroxidation, and the rigidity of muscle post-mortem. These factors are instrumental in the onset of foodborne pathogens, deterioration of organoleptic properties through malonaldehyde and volatile lipid compounds, and the diminution of nutritional value, as elucidated by Zavadlav et al. [39]. Consequently, extending the shelf life of fresh meat necessitates meticulous processing, packaging, and distribution to mitigate these challenges.

The palatability of meat, enhanced through cooking, owes to the transformation of intramuscular fats and moisture, which collectively augment flavor, juiciness, and tenderness. This culinary phenomenon is further enriched by the presence of branched-chain fatty acids, contributing to the gustatory experience. The process of cooking induces the breakdown of muscle tissues, including collagen and myofibrillar proteins, leading to a redistribution of moisture from the cellular to the interstitial spaces, thus altering the meat’s textural properties. This phenomenon has been empirically validated through the research findings of Ismail et al., who have elucidated these effects in two distinct studies [40,41].

Introduced in the 1970s, the SV technique revolutionized meat cooking by immersing the product in a water bath or steam environment, ensuring even heat distribution while minimizing flavor loss. This method, distinct from traditional cooking techniques, preserves the cellular structure of meat, thereby enhancing its textural and moisture retention characteristics, a process thoroughly studied by Kaur et al. [42] and fellow researchers Cui et al. [18]. The findings from these studies underscore the intricate relationship between meat’s structural properties and its culinary quality. Through advanced cooking methodologies like SV, it is possible to achieve a harmonious balance between flavor, texture, and nutritional integrity, thereby elevating the consumer’s gastronomic experience.

Different meat matrixes of different animal origins were subject to SV processing and will be detailed in the following, while the SV main effects are presented in Table 1.

**Poultry.** Sous-vide cooking, characterized by its precision in temperature control, has garnered attention for its efficacy in enhancing the quality of chicken breast. This method’s strategic application at temperatures of 55 °C and 65 °C, as explored by Noh et al. [37], for durations of 180 and 360 min, significantly elevates the meat quality by retaining moisture, thus yielding a tender and juicier texture. Furthermore, it induces a reduction in myoglobin redness, enhancing the visual appeal of the chicken, a key aspect of the culinary presentation. This observation is in harmony with findings from Park et al. [58], who demonstrated that SV cooking at 60 °C for 2 to 3 h markedly improves the sensory qualities by diminishing cooking loss and bolstering tenderness, further affirming SV’s role in maintaining meat integrity and augmenting sensory attributes.

In alignment with these studies, works by Kowalska et al. [22] underscore the superior water content and mass efficiency achieved through SV cooking compared to conventional methods. This advantage is pivotal for commercial cooking environments where efficiency and quality are paramount. The research conducted by Kerdpiboon et al. [59] on chicken breast corroborates the SV advantage, showcasing enhanced water-holding capacity, yield, and texture. The ideal cooking time, pinpointed at around 4 h, presents SV as a promising solution for rendering chicken, irrespective of the cut, more palatable and nutritious, thereby catering to dietary needs across diverse age groups.

The study by Haghighi et al. [43] delves into the significant impact of SV cooking conditions on moisture content, cooking loss, and lipid oxidation in chicken breast fillets, emphasizing the critical importance of fine-tuning SV’s parameters to attain the desired quality. This optimization is crucial in poultry preparation, where texture and moisture are paramount indicators of quality.

Further expanding on this notion, Hasani et al. [44] explored a novel two-step temperature SV process that enhances the texture and minimizes lipid oxidation in chicken breasts. This innovative approach to multi-stage SV cooking offers refined strategies for quality enhancement, equipping culinary professionals with advanced methodologies to maximize the sensory attributes of chicken.

Adding to the discourse, Haghighi et al. [43] specifically highlight how SV cooking conditions significantly influence the moisture content, cooking loss, and lipid oxidation in chicken breast fillets. This insight underscores the importance of meticulously optimizing SV’s parameters to achieve the pinnacle of quality, especially in poultry, where the key quality indicators are texture and moisture; such findings further the understanding of SV cooking’s potential, reinforcing the method’s versatility and effectiveness in enhancing the culinary qualities of chicken, making it an indispensable technique for culinary professionals aiming to achieve excellence in poultry dishes.

Similarly to chicken breasts, chicken thighs also benefit from SV cooking, exemplifying the method’s versatility across different meat cuts, as mentioned in studies by Noh et al. [37]. The uniform cooking and moisture retention, critical for preserving flavor and texture, are especially beneficial in meats traditionally considered tougher or more variable in texture, like chicken thighs. This advantage over traditional cooking methods, which often result in uneven cooking and moisture loss, is further supported by a study on the quality characteristics of SV chicken breast by Hasani et al. [60]. Their research emphasizes the importance of the combination of time and temperature in SV cooking for enhancing the water-holding capacity, texture properties, and juiciness of the meat, showing similar benefits can be expected for chicken thighs. The findings from both Noh et al. and Hasani et al. [37,60] highlight the SV method’s capacity to maintain meat integrity and improve sensory attributes across various cuts, making it a superior choice for achieving optimal flavor and texture in poultry.

The study by Song et al. [47] on chicken breast ham highlights SV cooking’s potential in health-conscious culinary practices, notably in reducing sodium intake without compromising sensory qualities. Their research found that reduced-salt SV-cooked chicken breast ham possesses comparable sensory qualities to the regular-salt conventionally cooked counterparts. This finding is pivotal in today’s health landscape, where reducing sodium intake is a critical public health objective. The significance of this study is further validated by the research conducted by Silva-Santos et al. [61], which explored the impact of innovative equipment to monitor and control salt usage during cooking at home on salt intake and blood pressure. Their work, although focusing on a different aspect of sodium reduction, complements Song et al.’s [47] findings by demonstrating practical strategies to reduce sodium intake, underscoring the importance of such dietary modifications in public health.

In the investigation led in 2023 by Wereńska et al. [45], the SV method was applied to goose breast, revealing an enhanced fatty acid profile that suggests significant nutritional benefits. This aligns with SV’s known capacity to improve both the flavor and health aspects of cooking, particularly evident in cooking with the skin, which often adds to the sensory appeal, thereby making SV an attractive method for preparing game and poultry meats. These findings further underscore the superiority of SV in preserving essential nutrients, including minerals and essential fatty acids, while minimizing undesirable changes such as lipid oxidation and nutrient loss [45]. This methodological advantage, coupled with the observed benefits in nutrient retention and cholesterol management, strongly positions SV cooking as a preferred choice for health-conscious individuals seeking to maximize the nutritional quality of goose meat without compromising on taste. Adding to this, the 2022 study by Thathsarani et al. [35] on the effects of SV cooking on the bio-functionality, nutritional value, and health benefits of salmon lipids support the notion that SV cooking not only preserves but potentially enhances the nutritional profile of meats, further highlighting SV’s role in health-conscious culinary practices.

In the study by Shin et al. [46], variations in cooking loss, color, and microbial content in SV-cooked duck breast were meticulously analyzed, showcasing the method’s adaptability and efficacy in cooking poultry. The study identified optimal SV cooking conditions that serve as valuable guidelines for both chefs and food processors, illustrating the method’s versatility in achieving culinary excellence in poultry preparation. This aligns with the findings of researchers Zhang et al. [62], which demonstrated that moderate cooking conditions are crucial for optimizing flavor and texture in SV duck meat. These conditions—specifically, moderate temperatures combined with precise cooking durations—were shown to significantly enhance the meat’s flavor and texture, emphasizing SV’s role in refining culinary practices for professionals. Additionally, the research subsequently conducted by Zhang et al. [63] on the impact of sodium chloride on the physicochemical and textural properties and flavor characteristics of sous-vide cooked duck meat further reinforces the importance of optimal cooking parameters. Their study evaluated the effect of salt brining on duck meat quality, underscoring how SV, coupled with the appropriate pre-treatment, can profoundly influence the flavor profile and overall quality of duck meat, thereby cementing SV cooking as a method of choice for those aiming to maximize the nutritional and sensory attributes of poultry without compromising on health aspects.

**Pork.** In the study by Yıkmış et al. [48], the low-temperature, long-duration cooking method of sous-vide is highlighted for its significant enhancement of the juiciness and nutrient concentration in Iberian pig meat. Here, preserving the nutritional content is as crucial as enhancing the taste, reflecting a broader culinary movement towards techniques that harmonize health benefits with gourmet standards. Further exploration into this subject by Belmonte et al. [64] in their 2022 study aligns with these findings. Belmonte et al. studied the physicochemical changes induced by SV, offering a comparative perspective on how different SV conditions can influence meat quality, potentially affecting its nutritional profile and sensory characteristics [64]. This finding underscores the nuanced potential of SV cooking to not only retain but possibly enhance the nutritional and sensory qualities of meats, offering a rich area for further culinary and scientific exploration.

In the research conducted by Cubon et al. [49], the SV method’s impact on pork shoulder was thoroughly examined, revealing significant alterations in both the fatty acid composition and phthalate content throughout the SV treatment process. These modifications highlight the method’s profound influence on the sensory and chemical attributes of the meat. Such insights are crucial for grasping the extent to which cooking techniques can affect the overall quality and safety of meat products. This conclusion is further substantiated by a comparative study conducted by Modzelewska-Kapituła et al. [65], which assessed the effects of microwave and SV cooking on the chemical composition, including the fatty acid composition of pikeperch fillets. Although focusing on a different type of meat, the findings from this study corroborate the notion that SV cooking can significantly influence meat’s fatty acid profile, thereby affecting its nutritional value and safety [65].

The SV cooking method’s potential in pork loin preparation is significantly amplified when combined with innovative pre-treatment techniques, as demonstrated by recent studies. Go et al. [50] explored the quality enhancement of SV pork loin through wet-aging, further intensified by employing a pulsed electric field system. This approach not only underscores the synergy between pre-treatment methods and SV cooking but also marks a transformative step in meat processing, potentially elevating both the sensory and nutritional qualities of the meat. This combination could indeed revolutionize the way pork loin is processed, offering a dual advantage of improved taste alongside the nutritional benefits.

On a related note, Wang et al. [51] shed light on the variability in lethality levels during the SV cooking of pork loin, emphasizing the paramount importance of precise control over SV’s parameters to ensure food safety. This insight is invaluable within the meat processing industry, where adhering to regulatory standards and safeguarding consumer health is of utmost importance. The variability noted by Wang et al. [51] serves as a crucial reminder of the intricate balance required in SV cooking to not only achieve the desired culinary outcomes but also meet rigorous food safety criteria. This finding becomes especially significant in the context of increasing consumer awareness and demand for safety in meat consumption, urging producers and culinary professionals to adopt meticulous SV parameter control to mitigate risks.

Together, the studies by both Go et al. and Want at al. [50,51] highlight the evolving landscape of pork loin preparation through SV cooking. While Go et al. [50] point towards the promising prospects of enhancing meat quality via combined pre-treatment and SV techniques, Wang et al. [51] caution the need for precision in SV parameter settings to ensure consumer safety. These insights collectively advocate for a nuanced approach to SV when cooking pork loin, balancing the pursuit of culinary excellence with the imperative of food safety.

**Other Meat Types.** The research by Stanisławczyk et al. [52] on horsemeat reveals how the SV cooking method excels in preserving the meat’s inherent color and minimizing weight loss, which are pivotal quality attributes for meats possessing unique flavors and textures, such as horsemeat. This characteristic retention is particularly valuable in specialty meat markets, where the preservation of distinct qualities is essential for upholding culinary authenticity. The study highlights SV’s potential in enhancing the appeal of specialty meats by maintaining their intrinsic properties, suggesting its applicability in markets that cater to niche culinary preferences. This approach is further supported by the findings in 2023 of Hobani et al. [11], which illustrate the effectiveness of SV and conventional electric oven cooking methods on the physio-sensory quality attributes of Arabian Camel meat. Though focusing on a different type of meat, this study corroborates the notion that SV cooking is adept at improving meat’s physical and sensory characteristics, making it a suitable cooking method for exotic and specialty meats where quality attributes such as tenderness, flavor, and moisture content are paramount [11]. Together, these studies underscore the versatility of SV cooking in preserving the quality and enhancing the sensory appeal of various types of meat, thereby positioning it as a valuable technique for culinary practices that prioritize the retention of unique meat characteristics.

**Beef.** In the study conducted by Karki et al. [36], increased soluble collagen and enhanced tenderness were observed in beef short ribs when cooked using the SV method. This indicates a notable transformation in the meat’s connective tissue, which is particularly beneficial for tougher cuts like short ribs. Such a transformation results in a texture that is more palatable, showcasing SV’s unique ability to break down collagen without drying out the meat—a common issue with traditional cooking methods. This advantage of SV is crucial for optimizing the sensory appeal of meats that require long cooking times to soften.

Expanding on these findings, the research by Yin et al. [66] provides insight into the mechanisms behind the tenderness improvement observed in beef treated with SV. Their study reveals that SV cooking significantly promotes the release of cathepsins B and L, enzymes responsible for protein degradation, from lysosomes. This enzymatic activity contributes to the breakdown of myofibrillar proteins and collagen, enhancing the meat’s tenderness. Furthermore, the study noted that SV-treated beef exhibited a higher myofibrillar fragmentation index, increased collagen solubility, and longer sarcomere length compared to traditionally cooked samples [66]. These biochemical changes underline the effectiveness of SV cooking in not only maintaining moisture but also in tenderizing meat by altering its protein structure.

Together, these studies [36,66] underscore the profound impact of SV cooking on improving the quality of beef, particularly in terms of tenderness and sensory characteristics. By facilitating a gentle and controlled cooking process, SV allows for the preservation of moisture and the enhancement of flavor, making it an invaluable method for culinary professionals and food enthusiasts alike.

In another analysis [9], SV cooking’s effectiveness in enhancing meat safety was underscored through its efficient inactivation of harmful pathogens like *L. monocytogenes* in beef tenderloin. This aspect of SV cooking, which prioritizes both safety and quality, is particularly pertinent in commercial food preparation settings, where adherence to health standards is of utmost importance. The method’s capacity to maintain the sensory qualities of beef tenderloin while achieving such safety benchmarks highlights its sophistication and appeal. Additional research emphasized SV’s role in preserving the nutritional quality of meats, demonstrating how the method minimizes the formation of harmful compounds [67]. This supports the SV method as a beneficial cooking technique for enhancing both the safety and nutritional profiles of meats, aligning well with health-conscious culinary practices.

Moreover, the studies on beef semimembranosus muscles [54] revealed that marinated beef semimembranosus muscles cooked using the SV method exhibited enhanced fatty acid composition, suggesting the method’s potential to amplify both flavor and nutrition. This aligns with broader culinary trends that aim to elevate taste while also boosting health benefits. The findings by Aviles et al. [68] on the impact of SV cooking on the nutritional quality of meat further fortify these observations, showcasing SV’s ability to retain natural sensory qualities and nutritional value, thereby making it an ideal choice for health-conscious cooking and commercial food preparation alike.

In the investigation by Kaur et al. [42], the SV cooking technique was shown to significantly improve the tenderness and texture of beef brisket, an insight for traditionally tough meat cuts. This method’s ability to tenderize and enhance the meat’s physical attributes underscores its utility in transforming less tender cuts into high-quality dishes. This is particularly vital for brisket, where conventional cooking methods can often lead to toughness [42].

Alahakoon et al. [53] further explored SV cooking, focusing on the optimization of temperature and time to enhance the tenderness and minimize cooking loss in beef briskets. Their findings align with the culinary movement towards utilizing more sustainable meat cuts by improving their taste and texture through innovative cooking techniques.

Moreover, the research by Gámbaro et al. [69] delved into how the adjustment of temperature and time in SV cooking affects the physicochemical and sensory parameters of beef shank cuts. While targeting a different beef cut, their insights confirm the SV method’s effectiveness in enhancing meat’s appeal and its physicochemical attributes. This parallel with brisket showcases SV cooking’s versatility and efficacy across various meat cuts, reinforcing its value in culinary practices aimed at elevating the quality of tougher meats.

In the 2019 study by Cosansu et al. [55], the addition of grapeseed extract to SV-cooked ground beef was found to reduce the heat resistance of *C. perfringens*, suggesting an innovative approach to enhancing food safety. This development aligns with the current culinary trends that prioritize both safety and health, illustrating SV’s capability to integrate food safety measures within its cooking process without compromising the sensory qualities of the meat. Similarly, in 2023, the research by Douglas et al. [56] on SV-cooked ground beef patties followed by grilling demonstrated a decrease in cook loss and color change, underscoring SV’s effectiveness in improving both texture and appearance. This dual-cooking method could prove especially beneficial in commercial kitchens where consistency and quality are paramount.

Further, the study on beef fingers by El-Badry et al. [57] revealed that SV cooking helps retain moisture, fats, and nutrients, indicating the method’s efficiency in boosting the nutritional and sensory properties of meat. This finding is particularly pertinent for culinary applications aimed at maximizing flavor and nutritional value. These investigations collectively highlight SV cooking’s role in enhancing meat’s physical and nutritional qualities while ensuring food safety.

Adding to this body of research, the study by Modzelewska-Kapituła et al. [70] on the nutritional value of cooked and SV beef emphasizes SV’s ability to preserve mineral compounds, thus maintaining the meat’s nutritional value. This work supports the notion that SV cooking not only improves the safety and sensory attributes of meat but also its nutritional profile. Additionally, the analysis by Berdigaliuly et al. [71] on the effects of SV cooking on semi-finished meat products further validates SV’s role in preserving meat juiciness and texture, providing a comprehensive understanding of SV cooking’s benefits across different meat types and preparations.

**Fish.** In the realm of SV cooking, particularly for *rainbow trout*, the incorporation of coriander essential oil as a natural antimicrobial agent has been investigated for its efficacy in mitigating the risk posed by *L. monocytogenes*. The study by Öztürk et al. [6] unveils the promising synergy between SV cooking and natural preservatives, such as coriander essential oil, in enhancing food safety. This fusion approach not only adheres to the growing consumer demand for natural food preservation methods but also elevates the microbial safety profile of SV-cooked foods.

Expanding upon these findings, subsequent research conducted by Zakrzewski et al. [72] delves into the specific challenges associated with the SV cooking of fish, particularly concerning the persistence of *L. monocytogenes*. Their investigation sheds light on the crucial observation that standard cooking temperatures commonly employed for fish do not necessarily guarantee the eradication of this pathogen. This revelation places a spotlight on the necessity of integrating SV cooking with potent antimicrobial agents, such as coriander essential oil, to safeguard against microbial threats. Zakrzewski et al.’s [72] work further articulates the critical need for meticulous cooking method selections and the adoption of additional safety protocols to ensure the microbiological integrity of SV culinary products.

These studies collectively highlight the nuanced interplay between cooking technology, natural antimicrobials, and food safety protocols. They underscore the imperative of leveraging both culinary innovation and antimicrobial efficacy to meet the dual objectives of sensory enhancement and microbial safety in SV cooking practices.

In their 2022 study, Modzelewska-Kapituła et al. [21] explored the SV cooking method’s capacity to preserve and amplify the nutritional qualities of pikeperch, notably its beneficial fatty acids, which are pivotal for health-conscious consumers. This research is particularly significant in the discourse on fish consumption, where the health benefits, especially those derived from omega-3 fatty acids, are a major attraction. The study delves into the distinctions between wild and farmed pikeperch, providing critical insights that could influence consumer preferences regarding fish products. It was found that SV cooking maintains the fatty acid profile of pikeperch fillets effectively, ensuring the conservation of valuable polyunsaturated fatty acids, including omega-3s, essential for a balanced diet. Moreover, the technique not only preserved the high sensory quality of the fillets, characterized by their favorable texture, aroma, and taste, but also enhanced the fat content when compared to microwave cooking. These findings, as reported in their subsequent 2023 study, affirm SV’s superiority as a cooking method for those aiming to optimize both the nutritional and sensory qualities of pikeperch, making it a prime choice for health-focused culinary professionals and consumers alike [65].

Further supporting this, the comparative analysis of microwave and SV cooking effects on the fatty acid composition and quality attributes of pikeperch fillets underscores the meticulous balance that SV strikes between enhancing the flavor and maintaining nutritional integrity [65]. This balance is crucial in the culinary industry, where the demand for methods that bolster both taste and health benefits is growing. Additionally, the exploration of muscle tissue quality in wild versus farmed pikeperch complements the broader understanding of how SV cooking can be tailored to different types of pikeperch to achieve desired health and sensory outcomes, further emphasizing the method’s adaptability and effectiveness in contemporary culinary practices [21].

In the domain of the SV cooking of salmon, researchers have raised pivotal considerations regarding the limitations of SV in achieving sufficient pasteurization for the effective reduction of pathogens, which is a critical aspect in ensuring food safety [35]. This underscores the imperative for culinary professionals and food processors to employ a combination of SV with other methods, such as freezing, particularly for seafood, which is notably susceptible to pathogens. This integrated approach is essential for mitigating food safety risks, especially with seafood’s inherent vulnerability to microbial contamination.

Complementing these insights, a further investigation has demonstrated that the incorporation of natural antimicrobials, specifically oregano oil and citric acid, can significantly amplify the thermal inactivation of *L. monocytogenes* in SV salmon [2]. This evidence accentuates the efficacy of integrating natural antimicrobials to bolster the safety profile of SV seafood, presenting a viable strategy for food processors aiming to elevate pathogen reduction measures in salmon.

These findings collectively advocate for the judicious selection of SV cooking conditions and the strategic use of natural antimicrobials to enhance the microbial safety of salmon. This approach aligns with the evolving culinary trends that prioritize both the sensory qualities and the health implications of food, thereby offering a comprehensive strategy for ensuring the safety and quality of SV seafood [2,35].

**Venison.** The lean and flavorful nature of venison presents a unique challenge for culinary endeavors. Traditional methods, susceptible to overcooking and inconsistent results, often fail to capture the full potential of this prized protein. The sous-vide method involves submerging vacuum-sealed venison cuts in a precisely regulated water bath, ensuring uniform doneness throughout the cut, regardless of the desired final temperature.

Beyond mere consistency, SV offers distinct advantages for venison preparation. Studies conducted have confirmed that SV produces significantly more tender and juicy venison steaks compared to conventional approaches [73]. This phenomenon can be attributed to meticulous temperature management, preventing overcooking, and preserving moisture within the lean muscle tissue. Furthermore, the vacuum-sealed environment fosters an intensification of natural flavors and aromas, as elucidated by Yin et al. [66]. Their research demonstrated that varying SV temperatures significantly influence the venison’s sensory profile, empowering chefs with precise control over the final taste and texture experience.

Recent research further supports the utility of SV in ensuring food safety alongside quality improvements in game meat preparation [74]. Their study, which focused on the inactivation of *L. monocytogenes* in game meat under SV cooking conditions, found that cooking temperatures between 50 °C and 60 °C effectively reduced the bacterial presence. Specifically, the study highlighted that the safe cooking duration for eliminating *Listeria* varies depending on the type of game meat, underlining the method’s adaptability to different culinary needs while emphasizing the importance of temperature control for food safety.

## 5. Sous-Vide in Vegetables Processing

The influence of SV on vegetables is equally noteworthy. It preserves the crispness, vibrant color, and natural integrity of vegetables like carrots, broccoli, and green beans, a feat that traditional boiling or steaming often fails to achieve. Sous-vide ensures that these vegetables are cooked thoroughly yet retain their nutritional content, making them both visually appealing and rich in essential vitamins and minerals. Thorough research exemplifies how SV elevates the texture, color, and nutrient profile of vegetables, enhancing their overall palatability [39,75].

Vegetables are reservoirs of numerous bioactive constituents, such as ascorbic acid, carotenoids, and phenolic entities, which are susceptible to alterations through various culinary techniques, including boiling, steaming, and frying. These modifications often lead to the degradation or oxidation of these bioactive components, which are otherwise bound within cellular matrices. Notably, the hydrophobic nature of carotenoids renders them less vulnerable to leaching during the culinary processes and storage, especially when vegetables are subjected to SV cooking. Research conducted by Guillén et al. [76] underscores the superior retention of antioxidant capabilities in vegetables like artichokes and carrots via SV cooking, in contrast to traditional boiling methods. This enhanced preservation extends to vital nutrients, where the SV method’s low-temperature and vacuum-sealed conditions minimize nutrient dissipation.

Further investigation into the effects of SV and the steaming of *Brassica* vegetables reveals a general decline in vitamin C across both methods, with steam cooking incurring more significant losses [77]. However, nutrient depletion is observed to be less pronounced in the stems compared to florets and leaves. Contrasting responses in phenolic content among different vegetable parts have been documented, with certain broccoli varieties exhibiting enhanced total phenolic content (TPC) when subjected to SV cooking, an effect not mirrored in the florets.

Florkiewicz et al. [78] demonstrate that SV cooking under specific conditions (90 °C for 45 min) optimizes the vitamin C content in broccoli, Romanesco, and Brussels sprouts, as opposed to the effects observed in cauliflower. Moreover, this method elevates the antioxidant activity compared to their raw counterparts, suggesting an enhancement or liberation of bioactive compounds under SV conditions. Additionally, different studies explore antioxidant activities across various vegetables subjected to both conventional and SV cooking, unveiling a diverse range of responses based on vegetable type and cooking method, with certain vegetables showing notable increases in antioxidant activity when cooked using the SV method [79].

The incorporation of natural extracts, such as rosemary essential oil, in SV cooking, has been explored by researchers [3,80,81], showcasing the potential for not only preserving but enhancing the nutritional and antioxidant profiles of vegetables through SV cooking. Moreover, contrasting findings from studies such as those by Rinaldi et al. [27] elucidate the nuanced effects of SV and other cooking methods on the retention and degradation of vital nutrients, emphasizing the significance of cooking method selection based on the desired nutritional outcomes. This compilation of research affirms SV cooking’s efficacy in maintaining and potentially augmenting the bioavailability of vital phytochemicals, aligning culinary practices with health-conscious consumption patterns.

An overview of the main effects of SV processing on vegetables is presented in Table 2 and will be detailed in the following paragraphs.

In the exploration of the impact of SV cooking on vegetables, Stanikowski et al. [75] conducted a study on carrots cooked at 80 °C and 90 °C. Their findings reveal that SV cooking notably improves the textural attributes of carrots, such as hardness, cohesiveness, and chewiness. This enhancement in texture highlights SV’s potential to elevate the sensory qualities of vegetables beyond those achieved through traditional cooking methods. Moreover, the study underscores SV’s capacity to preserve vital nutritional components, with a particular emphasis on carotenoids. The variant cooked at 90 °C for 10 min was especially effective, suggesting that SV cooking is adept at retaining nutrients that are typically diminished in conventional cooking processes.

Building upon these insights, Guillén et al. [76] further elucidate the nutritional advantages of SV cooking for carrots. Their research corroborates the enhanced preservation of carotenoids and phenolic content offered by SV, alongside a notable improvement in antioxidant activity retention—rising from a mere 9.2% with boiling to an impressive 55.3% with SV. These researchers [76] also observed that SV cooking more effectively maintains the color and visual appeal of carrots, thereby asserting SV as the superior method for preserving both the essential nutrients and sensory attributes of vegetables.

Together, these studies provide compelling evidence of SV cooking’s advantages over traditional methods [75,76]. They highlight the technique’s proficiency in not only enhancing the textural quality of vegetables like carrots but also in safeguarding their nutritional integrity and sensory appeal. This body of research underscores the value of SV cooking as an optimal culinary choice for maximizing the health benefits and sensory qualities of vegetables.

In research conducted by Stanikowski et al. [75], the impact of SV cooking on parsley was meticulously analyzed with an emphasis on both aesthetic and nutritional variables. The study delineates that SV cooking—particularly when contrasted against a boiling duration of 20 min—manifests a notable influence on the visual attributes of parsley, manifesting in a diminished brightness. This alteration in visual appeal highlights the intricate effects that SV cooking exerts on the sensory attributes of vegetables, potentially influencing consumer perception and acceptance.

Notwithstanding the visual modifications, the research delineates a significant advantage of SV cooking in the form of augmented retention of phenolic compounds within the treated parsley. This preservation of antioxidants underscores the efficacy of SV cooking in safeguarding and potentially enhancing the health-beneficial properties of vegetables. The sustained presence of phenolic compounds, renowned for their antioxidant capacities, is particularly consequential, given their indispensable role in promoting health benefits.

The investigative work by Stanikowski et al. [75] on the application of SV cooking to parsley offers a nuanced insight into the dualistic nature of this culinary technique. While it may alter certain sensory perceptions, such as visual appeal, it concurrently fortifies the nutritional profile by preserving key health-promoting compounds. This dual outcome underscores the potential of SV cooking as a valuable culinary strategy aimed at optimizing the health attributes of vegetables without detracting from their essential qualities, thereby contributing to the broader discourse on culinary science and nutrition.

In other research, SV cooking emerges as a superior method for preserving the inherent color, texture, and nutritional content of broccoli when compared to traditional cooking techniques [39]. This method’s ability to maintain the sensory and nutritional integrity of broccoli positions SV cooking as a preferred choice among health-conscious consumers and those seeking to preserve the natural attractiveness of vegetables. The effectiveness of SV in safeguarding these qualities underscores its utility in culinary practices focused on health and aesthetic presentation.

Complementing this, the study by Dos Reis et al. [83] provides further empirical support for SV’s advantages, demonstrating its capacity to significantly conserve higher levels of bioactive compounds in broccoli, such as flavonoids, carotenoids, and vitamin A, beyond what is achievable through boiling, steaming, or microwaving. By doing so, SV cooking not only secures the retention of broccoli’s vivid coloration and crisp texture, but it also secures its critical antioxidants, highlighting SV’s role in bolstering the nutritional consumption of vegetables cultivated organically. This research collectively accentuates the value of SV cooking in enhancing the dietary benefits derived from consuming organically grown vegetables, marking it as an effective method for optimizing the healthful properties of broccoli.

Sous-vide cooking has been identified as a highly effective technique for preserving the essential nutrients in green beans, with particular efficacy in retaining minerals at levels akin to those found in their raw state. This capability of SV to minimize mineral loss while also preserving the desirable color and consistency of green beans enhances its culinary appeal, making it a favored method among those seeking to maintain the nutritional and sensory qualities of vegetables. The 2023 study by Czarnowska-Kujawska et al. [82] underscores the significance of SV in the context of nutrient preservation, demonstrating its potential to offer cooked vegetables that closely mirror the nutritional profile of their uncooked counterparts, thereby underscoring the method’s utility in health-conscious culinary practices.

Sous-vide cooking, when applied to beetroots for extended periods, has been observed to result in a reduction in color intensity and consistency. Despite these alterations in sensory attributes, SV cooking stands out for its ability to significantly reduce the loss of dry mass, a common issue with other cooking methods. This aspect of SV cooking highlights its effectiveness in preserving the structural integrity and nutritional value of beetroots, making it a viable option for those aiming to retain the vegetable’s essential qualities. The research conducted by Czarnowska-Kujawska et al. [82] elucidates the nuanced impact of SV cooking on beetroots, showcasing its strengths in minimizing nutrient depletion while pointing to considerations regarding the vegetable’s aesthetic properties, thereby affirming SV’s role in optimizing the culinary and nutritional aspects of vegetable preparation.

The SV cooking technique has been identified as particularly effective for cauliflower, enhancing not just the vegetable’s distinct flavors but also its antioxidative capabilities post-processing. This dual benefit suggests that SV cooking excels in both augmenting the taste and health advantages of cauliflower, offering a compelling reason for its adoption in culinary practices focused on maximizing vegetable quality. The 2020 research conducted by Zavadlav et al. [39] initially highlighted SV’s potential in this regard, demonstrating its capacity to intensify cauliflower’s characteristic flavors while boosting its antioxidative potential. Complementing this, other studies further investigated the impact of SV on cauliflower’s phytochemical content [84]. Their findings indicate a significant preservation and enhancement of crucial phytochemicals, such as glucosativin, hydroxycinnamic acid derivatives, and kaempferol derivatives, when cauliflower is cooked using the SV method, contrasting sharply with a reduction of these beneficial compounds through boiling. This body of evidence collectively positions SV as an effective method for not only preserving but also enhancing the nutritional and sensory qualities of cauliflower, making it a preferred cooking technique for enhancing both the flavor and health benefits of this vegetable.

The innovative SV–MW (Sous-Vide–microwaving) technique has been identified as especially beneficial for the preparation of asparagus spears [72]. This method surpasses traditional cooking approaches in preserving the nutritive quality and color characteristics of asparagus, showcasing the synergistic potential of integrating SV with microwaving to achieve superior culinary outcomes. The research indicates that such a combination can significantly enhance the retention of essential nutrients and the visual appeal of certain vegetables, positioning SV–MW as a promising method for optimizing the sensory and nutritional aspects of asparagus spears.

The integration of SV cooking with the antimicrobial properties of essential oils, such as rosemary, oregano, and basil, offers a novel approach to preserving minimally processed potatoes, as revealed in the study by Zavadlav et al. [39]. This method not only enhances the flavor profile and extends the shelf life of potatoes by significantly inhibiting bacterial growth but also showcases the synergy between SV and natural preservatives in improving the quality and safety of vegetables like potatoes. Further insights from [85] into SV’s impact on potatoes show that it can alter the texture, color, and nutritional content comparably to traditional cooking methods. SV cooking notably softens potatoes by lowering the shear force and reducing resistant starch content to below 5%, demonstrating its effectiveness in achieving conventional cooking outcomes. This capability of SV, especially when used in conjunction with natural preservatives, underscores its potential for widespread application within the food industry, aiming to enhance both the sensory qualities and safety aspects of potato products.

## 6. Quality Parameters of SV-Processed Food

When coming to the quality parameters of the SV processed matrix, several aspects have been addressed in different studies, targeting the texture, color, nutrients, safety, tenderness, juiciness, water-binding ability, as well as cooking loss (Table 3).

One of the most significant advantages of SV cooking is its unmatched influence on texture. Texture is not just a sensory attribute; it is a gateway to our perception and enjoyment of food. It influences how we experience different foods, from the first bite to the process of chewing and swallowing. In SV cooking, the precise control of temperature allows for an unmatched manipulation of texture, gently breaking down fibers in meats and softening vegetables to the exact desired level.

In vegetables like carrots, parsley, broccoli, green beans, and beetroots, SV ensures an ideal balance between softness and firmness, a texture that traditional cooking methods often struggle to achieve, as noted in research by Zavadlav et al. [39]. The method’s low-temperature slow-cooking approach gently breaks down fibers without overcooking, preserving the natural integrity and palatability of vegetables. The same principle applies to meats, where SV transforms texture, tenderizing even the toughest cuts like beef short ribs, horsemeat, and chicken thighs, as highlighted by researchers in their studies [36,37]. This texture enhancement not only improves the eating experience but also makes SV an invaluable tool for both home cooks and professional chefs.

The SV method transforms the texture of vegetables like carrots, parsley, broccoli, green beans, beetroots, and celeriac into something that is often more palatable than their conventionally cooked counterparts. Researchers like Zavadlav et al. [39] and Stanikowski et al. [75] demonstrate how SV preserves the integrity of vegetables while making them softer and easier to consume. The technique achieves a balance, avoiding the mushiness often associated with overcooking while retaining a pleasant firmness. Specifically, the study on celeriac (*Apium graveolens* var. *rapaceum)* reveals that SV-treated products necessitated twice more chews and time for consumption and had the highest sample compression and shearing forces compared to the boiled and steamed samples [86]. This method consistently maintained the dominance of perceived firmness, regardless of cooking time, highlighting SV’s unique advantage in preserving the textural qualities of vegetables, providing a firmer and potentially more satisfying eating experience compared to traditional cooking methods.

The influence of cooking techniques on meat texture cannot be overstated. Investigations by Karki et al. [36] and Noh et al. [37], complemented by the findings of Zhu et al. [87], have demonstrated the remarkable capacity of SV to augment meat tenderness. This method, particularly when preceded by actinidin enzyme treatment, as elucidated by Zhu et al. [87], significantly refines the texture of challenging cuts such as beef brisket, transforming them into delectably tender servings. This approach not only maintains the essential characteristics of the meat but also elevates the sensory experience by enhancing the tenderness, moisture, and flavor profiles. Consequently, SV emerges as an exemplary culinary technique, furnishing chefs and consumers alike with a reliable means to achieve unparalleled consistency in meat tenderness, thereby redefining the culinary standards for meat preparation.

Color, a primary visual indicator of freshness and quality, is another quality parameter significantly impacted by SV cooking. The method’s gentle cooking process preserves the vibrant, natural colors of foods, a feature especially notable in vegetables. This preservation is not just aesthetically pleasing but often indicates the retention of nutrients, as color degradation in vegetables often accompanies nutrient loss.

The color of food is a visual cue that sets expectations regarding its freshness, flavor, and quality. In SV cooking, the gentle heat preserves the natural color of foods, which is often lost in more aggressive cooking methods.

The retention of vibrant colors in vegetables like carrots and broccoli is a significant advantage of SV [39,75]. This preservation not only enhances the visual appeal but can also be indicative of the retained nutritional value, as color degradation in vegetables often goes hand in hand with nutrient loss. Similarly, the 2022 study by Ilic et al. [86] on purple eggplant and zucchini further underscores the potential of SV in maintaining the natural coloration of vegetables. Their findings illustrate that SV can lead to a less pronounced browning on zucchini skin and affect eggplant flesh color in a method-specific manner, suggesting that SV cooking can influence the visual and aesthetic appeal of different vegetables, enhancing or preserving their natural colors and potentially improving consumer acceptance.

Latoch et al. [88] provide a clear example of SV cooking’s impact on meat, demonstrating its effectiveness in preserving the color and enhancing the texture of pork steaks marinated in dairy-based products like kefir, yogurt, and buttermilk. This study illustrates how the SV method—by maintaining a precise and controlled cooking environment—prevents the oxidation of myoglobin, thus preserving the meat’s natural vibrant color. Furthermore, this research highlights the technique’s ability to produce juicier, more visually appealing meat products, which aligns with consumer expectations for high-quality and aesthetically pleasing food [88]. The enhanced moisture retention characteristic of SV cooking contributes significantly to this outcome, showcasing the method’s superiority in culinary applications for improving the sensory qualities of meat. Through this example, the potential of SV cooking to meet and exceed modern culinary standards is underscored, advocating for its broader adoption in both professional kitchens and home cooking practices.

Regarding nutrients, SV excels in retaining essential vitamins, minerals, and antioxidants in foods. The cooking method’s ability to prevent nutrient leaching during the cooking process is a substantial advantage over traditional methods, where nutrients can be lost to cooking water or overheating. This aspect of SV cooking is particularly crucial in health-conscious culinary practices where preserving the nutritional value of ingredients is as important as flavor and texture. The preservation of nutrients during cooking is crucial for maintaining the health benefits of food. SV excels in this aspect, gently cooking the food and avoiding the leaching of water-soluble vitamins and minerals.

Research conducted by Zavadlav et al. in 2020 [39] and Stanikowski et al. in 2021 [75] underscores the profound impact of SV processing on vegetable quality, demonstrating that carrots, parsley, and broccoli retain higher levels of vitamins and antioxidants when cooked using the SV method compared to traditional methods, thereby enhancing both the nutritional profile and flavor of vegetables, aligning with consumer preferences for healthier and more appealing food options. Similarly, Thathsarani et al. [34] emphasize the benefits of SV processing in the meat industry, citing its ability to preserve sensory quality, inhibit lipid oxidation, and improve the shelf life by maintaining carefully controlled thermal conditions and a vacuum-sealed environment, enhancing meat tenderness without compromising the moisture content and offering consumers a nutritious and palatable dining experience. In summary, SV cooking represents a significant advancement in culinary science, preserving the nutritional integrity and sensory attributes of both meats and vegetables, meeting the demands of modern consumers for high-quality, nutritious food products, and heralding a new era of culinary innovation and healthier eating practices.

Sous-vide cooking markedly enhances the nutrient retention in meats, exemplified by its ability to preserve essential fatty acids, which are pivotal for a nutritious diet. This cooking method is especially beneficial for fish species like pikeperch, where it safeguards omega-3 fatty acids, crucial for health benefits [21,89], thus underscoring the significance of SV in meat preparation, particularly noting its impact on moisture conservation. Unlike conventional cooking methods that often result in nutrient depletion through moisture loss, SV cooking maintains the meat’s hydration. This is not merely advantageous for the sensory attributes of the meat, offering a juicier and more tender experience, but it is also crucial for retaining water-soluble vitamins and minerals. Their study further reveals that SV cooking substantially reduces lipid oxidation compared to traditional cooking methods. Lipid oxidation, detrimental to both the flavor and nutritional quality of meats, is mitigated in the controlled, low-temperature environment provided by SV. This preservation of lipids is critical for maintaining the healthful properties of fats, particularly essential fatty acids. Through these observations, Ayub et al.’s [89] research elucidates the paramount role of SV in modern culinary arts, highlighting its effectiveness in preserving the intrinsic nutritional value of meats while enhancing their palatability.

The safety aspect of SV cooking, particularly its efficacy in pathogen reduction, cannot be overstated. The precise temperature control allows for the effective inactivation of harmful microorganisms, making SV a safer cooking method, especially for meats like beef tenderloin and fish such as salmon. Studies such as those by Gál et al. [9] and Karki et al. [35] emphasize the method’s role in ensuring both the deliciousness and safety of the food.

Tenderness is a critical quality parameter for meat products. SV cooking is particularly effective in enhancing the tenderness of tough cuts of meat due to its low-temperature and long-time cooking approach. This method allows for the breakdown of collagen into gelatin without drying out the meat, resulting in a tender and juicy product. Studies have shown significant improvements in the tenderness of meats such as beef short ribs, horsemeat, and chicken thighs when cooked using SV methods [36,52].

Juiciness is another essential attribute that significantly affects the palatability of meat products. SV cooking helps retain the natural juices within the meat, preventing moisture loss that typically occurs in conventional cooking methods. This retention is due to the vacuum-sealing process and precise temperature control, which minimize the evaporation of water from the meat. Research indicates that SV-cooked meats, such as chicken breast and pork loin, exhibit higher juiciness levels compared to those cooked using traditional methods [37,51].

Water-binding capacity refers to the ability of meat to retain water during processing and cooking. SV cooking helps improve the water-binding capacity of meat, resulting in a more succulent and tender product. The gentle heat and vacuum environment prevent the loss of water-binding proteins, maintaining the meat’s hydration levels. This property is particularly beneficial for lean meats and poultry, which tend to lose moisture more easily [38,44].

Cooking loss is the reduction in the weight of food due to the loss of water and fat during cooking. SV cooking significantly reduces cooking loss compared to conventional methods. The vacuum-sealed bags prevent the evaporation of moisture, and the precise temperature control minimizes the loss of fat and water-soluble nutrients. Studies have shown that SV-cooked meats, such as beef brisket and pork loin, experience lower cooking losses, retaining more of their original weight and nutritional content [51,53].

Ensuring the safety of vegetables prepared using SV techniques necessitates a rigorous approach to temperature management and storage conditions to counteract the hazards associated with foodborne pathogens. As highlighted by Stringer et al. [90], the effective pasteurization of SV vegetables, such as heating at 70 °C for 2 min, can inactivate vegetative pathogens like *L. monocytogenes.* This strategy is critical for mitigating risks and is supported by predictive modeling. Additionally, the prevention of spore germination and toxin production by psychotropic spore-formers, specifically non-proteolytic *C. botulinum*, requires maintaining storage temperatures below 3 °C, emphasizing the vital role of controlled refrigeration. These measures, grounded in the insights provided by predictive microbiology and kinetic modeling, delineate a clear framework for producing safe, high-quality SV vegetables by accurately calibrating the cooking temperatures, durations, and refrigeration practices to effectively address the unique microbial risks posed by this cooking method.

For meats like beef tenderloin and salmon, SV cooking not only enhances the flavor and texture but also ensures food safety. Studies like those of both Redfern et al. (2021) and Gál et al. (2023) show the effectiveness of SV in reducing harmful bacteria to safe levels, which is a significant concern in meat preparation [9,35]. In summary, SV cooking, with its methodical and controlled approach, offers significant improvements in the texture, color, nutrient retention, and safety of a wide range of foods. Its ability to enhance the intrinsic qualities of ingredients, coupled with its versatility, makes SV a valued technique in modern culinary practices. As the culinary world continues to evolve, SV stands as a testament to the fusion of science and art in cooking, promising consistent results, enhanced flavors, and assured safety in every dish it prepares.

## 7. Benefits and Limitations of SV

Sous-vide cooking, distinguished by its meticulous temperature management, offers notable benefits over traditional methods, enhancing food’s nutritional value and shelf life. This technique ensures uniform cooking results, reducing labor costs due to its simplicity, and does not necessitate professional training for operation, thus facilitating its industrial application [24,91].

By utilizing vacuum-sealed packaging, SV cooking minimizes mineral loss, improving the bioavailability of nutrients, such as copper, calcium, potassium, iron, and magnesium, with particular efficacy observed in bovine liver studies [91]. Furthermore, the method preserves mineral content, enhancing nutrient digestibility and solubility [92] while also maintaining flavors, preventing moisture loss, and reducing the formation of harmful compounds.

Despite these benefits, concerns remain regarding the microbiological safety of SV-processed foods without additional treatments, highlighting the need for specialized equipment and training [93].

When coming to the benefits and limitations of SV’s uses in different environments, several aspects have been addressed in different studies, targeting domestic, gastronomic, and large-scale industrial use (Table 4).

Sous-vide, in domestic use, brings enhanced flavor and texture to meals, a factor particularly highlighted by [94]. The tight temperature regulation leads to perfectly cooked dishes, maximizing both taste and texture. Kosewski et al. [96] emphasize the nutritional retention aspect, highlighting SV’s ability to preserve vitamins and antioxidants in vegetables, which is often lost in traditional cooking methods. However, Helal et al. [95] point out the high equipment costs, which can be a barrier for many households. Additionally, the long cooking times, as noted by Wang et al. [51], may not suit the fast-paced lifestyle of many home cooks. The environmental concerns, especially the use of plastic bags, are raised by Mishra et al. [98], pointing out the need for sustainable practices in SV cooking.

In gastronomic kitchens, SV ensures consistent quality across servings, a significant advantage for maintaining standardization in dishes [31]. This consistency is critical in the restaurant industry, where customer satisfaction relies heavily on repeatable quality. Stankov et al. [31] discuss the culinary creativity and innovation that SV brings to professional kitchens, allowing chefs to experiment with flavors and textures. However, the initial cost of equipment [56] and space requirements [31] can be considerable for smaller establishments. Furthermore, the slow cooking process, as Wang et al. [51] note, may not align well with the fast-paced environment of some restaurants.

Sous-vide in large-scale industrial food processing enhances food safety, ensuring uniform cooking and reducing the risk of undercooked products, as noted by Snyder et al. [97]. This uniformity is crucial in large-scale food production where consistency is key. Sebastiá et al. [101] also highlight SV’s role in extending the shelf life of products, which is essential in the food industry. However, the high investment in equipment [97] and environmental concerns due to plastic use [98] are significant challenges.

Entities within the industrial sector have initiated the integration of avant-garde practices for the formulation of culinary creations that are not only elevated in nutritional content but are also prepared within stringent sanitary protocols. Within the spectrum of these methodologies, the SV culinary technique is distinguished as the paramount approach for engendering food items that excel in both technological and functional dimensions, as elucidated by de Boer et al. [102].

Sous-vide’s low-temperature cooking preserves the natural integrity of ingredients, resulting in dishes that are nutritious, delicious, and visually appealing. The method’s precision and efficiency can revolutionize cooking in various settings, though considerations regarding cost, cooking time, and environmental impact must be taken into account. As culinary technology evolves, the role of SV in professional kitchens and food processing facilities is likely to expand, driven by its unique advantages and the growing demand for high-quality, safe, and convenient food products.

## 8. Microbial Considerations in SV Products

Microbial safety is a critical concern in food processing and preparation, particularly for meat products, which are susceptible to contamination by pathogens. The SV cooking method offers several microbial safety benefits that enhance its appeal in both domestic and industrial settings.

Sous-vide cooking involves vacuum-sealing food in airtight bags and cooking it in a water bath at precisely controlled temperatures. Baldwin showed in his work in 2012 [103] how this precise temperature control is fundamental in achieving pasteurization, effectively reducing the microbial load while preserving the food’s nutritional and sensory qualities. The low and consistent temperatures used in SV cooking (generally between 50 °C and 85 °C) ensure that harmful bacteria are eliminated without the risk of overcooking, which is common in traditional methods.

Studies such as those by Gál et al. [9] have demonstrated the effectiveness of SV cooking in inactivating *Listeria monocytogenes* in beef tenderloin. Similarly, Redfern et al. [35] highlighted the antithrombotic properties and reduced lipid oxidation in SV-cooked salmon, emphasizing the method’s role in ensuring food safety while maintaining quality.

Sous-vide cooking’s effectiveness in pathogen reduction is well-documented. The method is particularly adept at eliminating pathogens, such as *Salmonella*, *Escherichia coli*, and *Clostridium perfringens*, which are commonly associated with meat products. For instance, the research conducted by Dogruyol et al. [2] showed increased thermal sensitivity of *Listeria monocytogenes* in SV salmon when oregano essential oil and citric acid were incorporated, demonstrating the method’s flexibility in integrating natural antimicrobials to enhance safety.

The vacuum-sealing process used in SV cooking not only enhances the flavor and texture but also extends the shelf life of meat products. By eliminating oxygen, SV significantly reduces the risk of aerobic bacterial growth. This was evidenced in the study by Abel et al. [74], which found that SV cooking conditions were effective in inactivating *Listeria monocytogenes* in game meat, thereby improving its safety and extending its shelf life.

The integration of natural preservatives such as essential oils in SV cooking further enhances its microbial safety profile. Essential oils from thyme, rosemary, and sage have shown significant antimicrobial effects against *Listeria monocytogenes* and other pathogens in meat products. For example, Gouveia et al. [8] demonstrated that sage essential oil was effective as an antimicrobial agent in SV beef during storage, highlighting a promising approach to natural food preservation. Furthermore, to further reinforce this, recent studies by Ciotea et al. [104] and Marchidan et al. [105] have shown similar antimicrobial benefits of essential oils in food preservation.

For the meat processing industry, SV cooking offers a reliable method for ensuring the microbial safety of products without compromising quality. Industrial applications of SV technology, as discussed by Haux [32], involve sophisticated vacuum packaging machines and large-scale water baths, ensuring uniform cooking and pathogen reduction across extensive quantities of meat. This consistency is crucial for meeting food safety standards and consumer expectations.

## 9. Conclusions

Our study confirms that sous-vide cooking enhances the flavor and texture consistencies significantly while preserving the nutritional integrity of the food. These outcomes align with previous studies, highlighting sous-vide’s ability to maintain controlled cooking environments, thereby reducing nutrient degradation and ensuring uniform cooking results [79]. However, the evidence does have limitations due to the variability in study designs and the specificity of sous-vide settings (time, temperature, and packaging) across different studies. This variability can affect the generalizability of the results. Additionally, many studies focus on specific types of food, such as meats or certain vegetables, which might not universally represent all possible sous-vide applications.

One limitation is the potential for publication bias, as studies with positive outcomes are more likely to be published than those with negative or neutral results. Despite efforts to include gray literature and unpublished studies, this bias might still impact the overall findings. Furthermore, the difficulty of including non-English language studies could omit relevant international research, potentially skewing the interpretation of sous-vide’s global applicability and benefits.

The implications of our findings are significant for both practice and policy. For culinary professionals and the food service industry, the results endorse sous-vide as a method that not only enhances food quality but also contributes to food safety by reducing the likelihood of pathogen presence when properly managed. For policymakers, these safety and nutritional benefits present a case for promoting sous-vide cooking in commercial settings as a way to improve public health outcomes. However, environmental concerns regarding the use of plastic in sous-vide cooking suggest a need for policy development focused on sustainable practices within the technique’s application.

**Future research** should aim to address the variability in study designs and sous-vide settings to enhance the generalizability of the results. Expanding the scope of studies to include a wider variety of food types beyond meats and certain vegetables will provide a more comprehensive understanding of sous-vide’s applications. Additionally, efforts to mitigate publication bias and include non-English language studies are crucial for a more accurate and global perspective on the benefits and limitations of sous-vide cooking.

Investigating alternative, sustainable materials for sous-vide packaging could address the environmental concerns associated with plastic use. Further research should also explore the long-term health impacts of sous-vide cooking and its potential role in public health nutrition strategies. By addressing these areas, future studies can provide more robust evidence to support the integration of sous-vide cooking into both culinary practice and public health policy.

In conclusion, sous-vide cooking presents numerous advantages in terms of flavor, texture, and nutritional integrity, reaffirming its value within the culinary arts and food service industries. However, to fully harness its potential, further research addressing the current limitations and environmental concerns is necessary. This will not only bolster the evidence base but also guide policy development for more sustainable and health-conscious applications of sous-vide cooking. The integration of these findings into broader culinary practices and public health strategies underscores the importance of continued investigation and innovation within this field. By advancing our understanding and application of sous-vide, we can enhance our food quality, safety, and sustainability for diverse populations worldwide.

## Figures and Tables

**Table 1:** Effects of SV cooking on various meats and fish.

Meat Type	SV Condition	Key Findings	Reference
Chicken Breast	SV at 55 °C and 65 °C for 180 min and 360 min	Enhanced lightness, reduced myoglobin redness, lower cooking loss	[37]
Chicken Breast	SV at 65 °C for 24 min	Higher water content and mass efficiency in SV-cooked chicken breast	[22]
Chicken Breast fillet	SV at 60, 70, and 80 °C for 60 min, 90 min, 120 min, and 150 min	Variations in moisture content, cooking loss, and lipid oxidation at different SV temperatures	[43]
Chicken Breast	SV at 50 °C and 60 °C for 120 min	Two-step temperature SV showed improved texture and decreased lipid oxidation in chicken breast	[44]
Chicken Thighs	SV at 55 °C and 65 °C for 180 min and 360 min	Similar effects to chicken breast under SV conditions	[37]
Goose Breast	SV at 70 °C for 4 h	Improved fatty acid profile, better cooking benefits with skin	[45]
Duck Breast	SV at 50, 60, 70, or 80 °C for 60 and 180 min	Variations in cooking loss, color, and microbial content based on cooking temperature and time	[46]
Chicken Breast Ham	SV at 60 °C for 2 h	Reduced-salt and SV cooking comparable to regular-salt and conventionally cooked chicken breast ham	[47]
Iberian Pigs	SV between 60 °C and 80 °C for 5–12 h	Juicier meat, higher nutrient concentration	[48]
Pork Shoulder	SV at 50 °C and 60 °C for 4 h and 8 h	Changes in fatty acid composition and phthalate content during SV treatment	[49]
Pork Loin	SV at 65 °C for 120 min	Wet-aging using a pulsed electric field system improved multiple quality-related properties of SV pork loin	[50]
Pork Loin	SV at 60 °C for 31 min and 41 min	Variation in lethality levels during SV, indicating different degrees of pasteurization	[51]
Horsemeat	SV at 55 °C, 60 °C, and 65 °C for 4–24 h	Lower weight loss at lower temperatures, better color retention	[52]
Beef Briskets	SV at 60 °C, 65 °C, and 70 °C for 24 h, 48 h, and 72 h	Sous-vide temperature and time optimization for improved tenderness and cooking loss in beef briskets	[53]
Beef Brisket	SV at 50 °C, 55 °C, 60 °C, 65 °C, and 70 °C for 1, 5, or 24 h	Cathepsins B and L contribute to improved meat tenderness in SV-cooked brisket	[42]
Beef Semimembranosus Muscles	SV at 60 °C for 4 h	Marinading prior to SV improved fatty acid composition in beef	[54]
Beef Tenderloin	SV at 55 °C, 60 °C, and 65 °C for 50 min	Effective inactivation of *L. monocytogenes*, enhanced safety	[9]
Beef Short Ribs	SV at 60 °C, 65 °C, and 70 °C for 12–36 h	Increased soluble collagen and tenderness at higher temperatures	[36]
Ground Beef	SV at 23 °C to 75 °C for 60 min	Reduced heat resistance of *C. perfringens* with grapeseed extract addition in SV-cooked ground beef	[55]
Ground Beef Patties	SV for 30 min, 60 min, or 90 min, then grilled	Decreased cook loss and change in cooked color with increased SV cooking time	[56]
Beef Fingers	SV at 85 °C for 4 h	Improved retention of moisture, fats, and nutrients in SV beef fingers	[57]
Salmon	SV at 50 °C for 20 min	Requires freezing for pathogen reduction; pasteurization challenges	[35]
Pikeperch	SV at 65 °C for 40 min	Better chemical and fatty acid compositions in wild pikeperch	[21]
Rainbow Trout	SV at 50 °C and 55 °C	Effective inactivation of *L. monocytogenes* with coriander essential oil in SV trout fillets	[6]

**Table 2:** Effects of sous-vide cooking on various vegetables.

Vegetable	SV Condition	Key Findings	Reference
Carrots	SV at 80 °C and 90 °C for 10, 20, 30 min	Higher hardness, cohesiveness, and chewiness in SV 80 °C variants. Highest carotenoid retention in SV 90 °C for 10 min.	[75]
Parsley	SV at 80 °C and 90 °C for 10, 20, 30 min	Lower brightness in boiled 20 min treatment. Highest retention of phenolic compounds in boiled 20 min treatment.	[75]
Broccoli	SV at various temperatures and times	Preservation of color, texture, and nutrients compared to conventional methods; retains more aroma and taste than conventionally cooked samples.	[39]
Green Beans	SV at 85 °C for 30 min, 60 min, 90 min	Sous-vide retains minerals at levels comparable to raw vegetables. Reduces loss of minerals and preserves desired color.	[82]
Beetroots	SV at 85 °C for 45 min, 90 min, 180 min	Longer SV processing times led to lower color intensity and consistency and minimized dry mass loss compared to other cooking methods.	[82]
Cauliflower	SV at various temperatures and times	Sous-vide intensifies characteristic flavors, enhancing antioxidative potential after processing.	[39]
Asparagus Spears	SV at various temperatures and times	SV-MW method was found to be most suitable for preserving nutritive quality and color.	[39]
Potatoes	SV with rosemary, oregano, basil essential oil	Combined use of REO and vacuum packaging controls the growth of bacteria in minimally processed potatoes, enhances flavor, and prolongs shelf life.	[39]

**Table 3:** Sous-vide cooking effects on quality parameters.

Parameter	Matrix	Reference
Texture	carrots	[75]
Texture	parsley	[75]
Texture	broccoli	[39]
Texture	green beans	[82]
Texture	beetroots	[82]
Texture	chicken breast	[37]
Texture	beef short ribs	[36]
Texture	horsemeat	[52]
Texture	chicken thighs	[37]
Texture	Iberian pigs	[48]
Texture	beef tenderloin	[9]
Texture	salmon	[35]
Texture	goose breast	[45]
Texture	pikeperch	[21]
Color	carrots	[75]
Color	parsley	[75]
Color	broccoli	[39]
Color	green beans	[82]
Color	beetroots	[82]
Nutrients	carrots	[75]
Nutrients	parsley	[75]
Nutrients	broccoli	[39]
Nutrients	cauliflower	[39]
Nutrients	potatoes	[39]
Nutrients	pikeperch	[21]
Safety	beef tenderloin	[9]
Safety	salmon	[35]
Tenderness	beef short ribs	[36]
Tenderness	horsemeat	[52]
Tenderness	chicken thighs	[37]
Juiciness	chicken breast	[37]
Juiciness	Iberian pigs	[48]
Water binding	chicken breast	[38]
Water binding	lean meats	[44]
Cooking loss	beef brisket	[53]
Cooking loss	pork loin	[51]

**Table 4:** Benefits and limitations of SV use in different environments.

Use	Benefits	Limitations	References
Domestic	Enhanced flavor and texture [94]	High equipment cost [95]	[94,95]
	Nutrient retention [96]	Long cooking times [51]	[51,96]
	Food safety [97]	Plastic waste [98]	[97,98]
	Convenience and ease of cooking [28]	Skill and knowledge required [31]	[28,31]
	Consistent cooking results [99]	Energy consumption concerns	[99]
Gastronomic	Consistent quality across servings [31]	Initial cost of equipment [56]	[31,56]
	Culinary creativity and innovation [31]	Space requirements for equipment	[31]
	Efficiency in preparation [94]	Slow cooking process [51]	[51,94]
	Extended shelf life of dishes [97]	Additional steps for texture [100]	[97,100]
	Reduced food waste [1]	Staff training needed [31]	[1,31]
Industrial	Uniform cooking for large batches [97]	High investment in equipment	[97]
	Enhanced food safety [101]	Large space requirement	[101]
	Extended product shelf life [97]	Environmental impact of plastic use [98]	[97,98]
	High-quality ready-to-eat meals [1]	Specialized training for staff [97]	[1,97]
	Scalability in food production [97]	Energy-intensive for large scales [99]	[97,99]

## Data Availability

No new data were created or analysed in this study. Data sharing is not applicable to this article.
